# Genetic Variants in Genes of the Inflammatory Response in Association with Infective Endocarditis

**DOI:** 10.1371/journal.pone.0110151

**Published:** 2014-10-09

**Authors:** Melanie Weinstock, Imke Grimm, Jens Dreier, Cornelius Knabbe, Tanja Vollmer

**Affiliations:** Institut für Laboratoriums- und Transfusionsmedizin, Herz- und Diabeteszentrum Nordrhein-Westfalen, Universitätsklinikum der Ruhr-Universität Bochum, Bad Oeynhausen, Germany; Sudbury Regional Hospital, Canada

## Abstract

**Aims:**

Inflammation in infective endocarditis (IE) is a complex network including interactions of inflammatory cytokines and other components of host response. Certainly, any variation in this network could influence susceptibility or disease progression of IE. In this study, 14 single nucleotide variants (SNVs) in genes coding for interleukin-1β, interleukin-6, interleukin-10, toll–like receptor-4, tumor necrosis factor-α, selectin E and intercellular adhesion molecule-1 were analyzed for an association with susceptibility to IE and correlated with disease-related laboratory parameters. Furthermore, the occurrence of SNVs was examined to elucidate pathogen-dependent associations.

**Methods and Results:**

The distribution of SNVs was determined in IE-patients and healthy blood donors by RFLP analysis. White blood cells (WBC) were counted using flow cytometry, concentration of C-reactive protein and procalcitonin was measured immunologically. Interleukin-6 c.471+870G>A genotypes differed significantly between IE patients and controls. The frequency of the heterozygote genotype GA was considerably higher in the patient group (68.9% vs. 43.8%, P_c_<0.0003). Interleukin-6 c.-237 minor allele frequency was increased in patients, although not statistically significant. Additionally, we detected a potential relation between interleukin-1β c.315C>T and IE. Pathogen-dependent analysis showed no significantly associated subgroup in relation to IE susceptibility, but gave hints towards alterations regarding *Enterococcus*-caused IE cases. Patients with genotype selectin-E c.-19 GT tend to have higher preoperative WBC counts than patients with genotype GG. We further showed an association between two interleukin-1β SNVs and laboratory biomarkers.

**Conclusion:**

This study shows genetic predispositions for the establishment of IE. Furthermore, correlation of SNVs with disease-related biomarkers suggests a role of genetic variants regarding the inflammatory response in IE.

## Introduction

Infective endocarditis (IE) is a microbiological infection of the endothelial lining of the heart valves with a high morbidity and mortality [1], [2]. The spectra of causing organisms is widely spread throughout gram-positive and gram-negative bacteria, but the gram-positive genera *Streptococcus*, *Staphylococcus* and *Enterococcus* represent the primary cause of IE cases [3], [4].

The pathophysiology of IE represents a multifactorial process, in which several different components determine the extent and outcome of IE. Initially, bacteria have to enter the bloodstream, followed by transport to endocardial structures, where bacteria have to adhere to and/or invade endothelial cells [5]. In this context, the inflammatory cascade in response to the causative microorganisms is crucial for disease progression. Variations of single inflammatory parameters could influence the host defense mechanism and the establishment of IE could be facilitated throughout the dysfunctional equilibrium [6], [7]. It has been shown for sepsis and other bloodstream infections that the occurrence of single nucleotide variants (SNVs) can influence the infection process, whereas information about SNVs in association with IE is rare [8].

Pathogen recognition could be altered with distinct genetic variants in genes coding for toll-like receptors or other recognition structures. A SNV in the gene coding for human toll-like receptor 2 was shown to be associated with increased risk for IE [9]. Additionally, two potentially associated SNVs of the lipopolysaccharide-binding protein were identified to influence IE susceptibility [10]. In contrast, SNVs in genes coding for platelet receptors GPIIIa and FcyRIIa were not associated with the risk of IE cases [11]. The cytokine host response includes several candidate genes, where SNVs could influence the inflammatory host response [8]. Several studies showed an association of tumor necrosis factor-α (TNF) or interleukin (IL)-6 SNVs with outcome of septic patients [12]. Adhesion molecules like selectin E (SELE) or intercellular adhesion molecule-1 (ICAM1) showed an importance in IE in the course of endothelial activation, through their high expression on infected heart valves [13]. Additionally, a SNV in SELE showed an association with altered monocyte adhesion or procoagulant activity in response to human endotoxemia [14], [15].

As previously shown, laboratory parameters of IE patients can give hints towards disease progression or extent. For example, Wallace et al. showed a relation of abnormal white blood cell (WBC) counts and increased mean C-reactive protein (CRP) concentrations with the mortality of IE patients [16]. Cornelissen et al. revealed that procalcitonin (PCT) in IE patients could serve as a possible biomarker for the disease outcome [17]. Variations of inflammatory parameters as a consequence of SNVs in the corresponding genes could possibly be shown by abnormal biomarkers. Therefore, an influence of SNVs on IE disease progression or outcome could be shown by abnormal laboratory infection parameters.

To point out potential associations of genetic variations to IE, we evolved an association study which includes 14 SNVs in genes involved in the inflammatory response to IE. For this purpose, we selected genetic variants of different proteins involved in the whole infection process (pathogen-recognition, early inflammatory response, inflammatory signaling, and restriction of inflammatory response). Particular SNVs were in discussion regarding an association for sepsis (e.g. TNF, IL6, IL10) or having an potential bias to pathobiochemical processes of inflammation (e.g. SELE) [8], [15]. For the first time, the patient group was divided into several pathogen-related subgroups (*Staphylococcus*, *Streptococcus*, *Enterococcus* and others) to study a pathogen-dependent association. Finally, we examined data regarding inflammatory laboratory parameters (CRP, WBC, PCT) to find out whether there are hints towards an altered inflammatory response as a consequence of SNVs.

## Methods

### Study design and cohort characteristics

The investigation was a retrospective study concerning the genotypes of 14 SNVs in IE patients, which were hospitalized in the heart and diabetes center NRW (Bad Oeynhausen, Germany) and underwent surgical intervention in course of IE. For examination of genotypes of SNVs in this study, only back-up samples of DNA extracted from surgically removed heart valves were available. The concentration of genomic DNA in patient samples varies due to the heterogeneous character of surgically removed heart valves. Furthermore, the availability of tissue material for DNA extraction was limited, therefore not all patients were analyzed for each SNV. Identification of causative pathogens was performed by standard blood culture methods or broad-range bacterial PCR of surgically removed heart valves, as described previously [4]. The control cohort consisted of 185 healthy blood donors. The characteristics of patients and controls are summarized in the Table S1. The study protocol was approved by the institutional review board of the Ruhr University of Bochum and all patients provided informed written consent.

### Sample preparation

EDTA-anticoagulated whole blood was collected in EDTA monovettes (Sarstedt, Nümbrecht, Germany). Serum samples were collected in serum monovettes (Sarstedt), followed by clotting reaction and centrifugation (4000×g, 10 min). Within two days of collection, samples were stored at −70°C until assayed as described below. Genomic DNA of IE patients was isolated from surgically removed heart valves. Total DNA was extracted with the QIAamp DNA blood kit (Qiagen, Hilden, Germany) according to the manufacturer’s instructions, and DNA was eluted with 50 µL sterile distilled water from the QIAamp column.

### RFLP analysis of genetic variants

The genetic variations analyzed in this study were genotyped by restriction fragment length polymorphism (RFLP) analysis. PCR primers and restriction enzymes are listed in the Table S2. Each 25 µL PCR reaction contained HotMaster Taq PCR buffer with 1.5–3.5 mM MgCl_2_ (Table S2), 200 nM of each dNTP, 2 µM of each primer and 1.25 U HotMaster Taq DNA polymerase (5Prime, Gaithersburg, USA). Thermal cycling conditions were as follows: initial denaturation at 95°C for 300 s, followed by 45 amplification cycles (denaturation at 95°C for 30 s; annealing for 30 s [temperature according to Table S2]; amplification at 72°C for 60 s). Restriction of PCR products was performed for 16 h according to manufacturer’s instructions for each enzyme (Table S2). PCR products were digested with restriction endonucleases and analyzed by agarose gel electrophoresis.

### Measurement of CRP, PCT and WBC counts in sera of IE patients

WBC counts were measured immediately after collection of EDTA-anticoagulated blood using the Cell-dyn Ruby (Abbott Diagnostics, Illinois, USA). CRP concentration in serum samples of IE patients was measured with the ultrasensitive latex immunoassay CRP Vario (Abbott Diagnostics) and PCT measurement was performed using the immunoluminometric assay on the LIAISON analyzer (DiaSorin, Stillwater, USA), according to manufacturer’s instructions.

### Statistical analysis

All gene variants were tested for confirmation with the Hardy-Weinberg equilibrium. Allele frequencies between cases and controls were compared using the Fisher’s exact test (Odds ratio and 95% CI). P-values were additionally proved using the Bonferroni method. Genotypes were compared using the χ^2^-test. Analysis was performed using GraphPad Prism 5.0 software (GraphPad, San Diego, USA). Haploview 4.2 was used for determination of haplotypes, whereas frequencies were compared using the χ^2^-test. CRP levels, PCT levels and WBC counts were compared using the Mann-Whitney test. P-values<0.05 were considered to be significant.

## Results

### Association analysis of sequence variations

The allelic distribution of the 14 analyzed SNVs is listed in Table 1. All analyzed frequencies were in the Hardy-Weinberg equilibrium. For TLR4, SELE, ICAM1 and IL10 genetic variants included in this study, no noticeable differences were observed. Although statistical significant differences were solely shown for IL6 c.471+870G>A genotypes, some noticeable varieties, regarding the other tested genetic variants in IL1B, TNF and IL6, were observed.

**Table 1:** Allele and genotype frequencies of IL1B, TLR4, SELE, ICAM1, IL10, TNF and IL6 gene variants in IE patients and healthy controls (**P_χ:_** P-value of χ^2^ test; P_F_: P-value after Fisher’s exact test; P_c_: corresponding P-values after Bonferroni correction).

Variation	Group	Genotype frequencies [n(%)]				Allele frequencies [n(%)]			
		**TT**	**TC**	**CC**	**P_χ_ (P_c_)**	**T**	**C**	**Odds ratio (95% Cl)**	**P_F_ (P_c_)**
**IL1B**	**IE patients**	46 (44.2)	51 (49.1)	7 (6.7)	0.934	143 (68.7)	65 (31.3)	0.9429	0.778
**c.-118C>T**	**controls**	86 (46.5)	87 (47.0)	12 (6.5)	(1.000)	259 (70.0)	111 (30.0)	(0.6525 to 1.362)	(1.00)
		**GG**	**GA**	**AA**	**P_χ_ (P_c_)**	**G**	**A**	**Odds ratio (95% Cl)**	**P_F_ (P_c_)**
**IL1B**	**IE patients**	44 (42.3)	54 (51.9)	6 (5.7)	0.124	142 (68.3)	66 (31.7)	0.7814	0.204
**c.302-64G>A**	**controls**	72 (38.9)	88 (47.6)	25 (13.5)	(0.372)	232 (62.7)	138 (37.3)	(0.5451 to 1.120)	(0.612)
		**CC**	**CT**	**TT**	**P_χ_ (P_c_)**	**C**	**T**	**Odds ratio (95% Cl)**	**P_F_ (P_c_)**
**IL1B**	**IE patients**	45 (43.3)	51 (49.0)	8 (7.7)	0.043	141 (67.8)	67 (32.2)	1.3370	0.149
**c.315C>T**	**controls**	105 (56.8)	63 (34.1)	17 (9.1)	(0.129)	273 (73.8)	97 (26.2)	(0.9220 to 1.940)	(0.447)
		**AA**	**AG**	**GG**	**P_χ_ (P_c_)**	**A**	**G**	**Odds ratio (95% Cl)**	**P_F_ (P_c_)**
**TLR4**	**IE patients**	127 (85.8)	21 (14.2)	0 (0.0)	0.248	275 (92.9)	21 (7.1)	1.411	0.326
**c.776A>G**	**controls**	167 (90.3)	17 (9.2)	1 (0.5)	(0.496)	351 (94.9)	19 (5.1)	(0.7435 to 2.677)	(0.652)
		**CC**	**CT**	**TT**	**P_χ_ (P_c_)**	**C**	**T**	**Odds ratio (95% Cl)**	**P_F_ (P_c_)**
**TLR4**	**IE patients**	127 (85.8)	21 (14.2)	0 (0.0)	0.274	275 (92.9)	21 (7.1)	1.411	0.290
**c.1076C>T**	**controls**	166 (89.7)	19 (10.3)	0 (0.0)	(0.548)	351 (94.9)	19 (5.1)	(0.7435 to 2.677)	(0.580)
		**GG**	**GT**	**TT**	**P_χ_ (P_c_)**	**G**	**T**	**Odds ratio (95% Cl)**	**P_F_ (P_c_)**
**SELE**	**IE patients**	120 (80.5)	29 (19.5)	0 (0.0)	0.415	269 (90.3)	29 (9.7)	0.7593	0.272
**c.-19G>T**	**controls**	140 (75.7)	44 (23.8)	1 (0.5)	(0.415)	324 (87.6)	46 (12.4)	(0.4642 to 1.242)	(0.272)
		**AA**	**AG**	**GG**	**P_χ_ (P_c_)**	**A**	**G**	**Odds ratio (95% Cl)**	**P_F_ (P_c_)**
**ICAM1**	**IE patients**	47 (30.9)	77 (50.7)	28 (18.4)	0.519	171 (56.2)	133 (43.8)	0.9582	0.815
**c.1405A>G**	**controls**	54 (29.2)	104 (56.2)	27 (14.6)	(0.519)	212 (57.3)	158 (42.7)	(0.7054 to 1.302)	(0.815)
		**GG**	**GA**	**AA**	**P_χ_ (P_c_)**	**G**	**A**	**Odds ratio (95% Cl)**	**P_F_ (P_c_)**
**TNF**	**IE patients**	97 (92.4)	9 (7.6)	1 (0.9)	0.335	203 (94.9)	11 (5.1)	1.378	0.525
**c.-418G>A**	**controls**	171 (92.4)	14 (7.6)	0 (0.0)	(1.000)	356 (96.2)	14 (3.8)	(0.6139 to 3.093)	(1.000)
		**GG**	**GA**	**AA**	**P_χ_ (P_c_)**	**G**	**A**	**Odds ratio (95% Cl)**	**P_F_ (P_c_)**
**TNF**	**IE patients**	75 (70.1)	27 (25.2)	5 (4.7)	0.416	177 (82.6)	37 (17.4)	0.7826	0.282
**c.-488G>A**	**controls**	116 (62.7)	60 (32.4)	9 (4.9)	(1.000)	292 (78.9)	78 (21.1)	(0.5072 to 1.207)	(0.846)
		**CC**	**CA**	**AA**	**P_χ_ (P_c_)**	**C**	**A**	**Odds ratio (95% Cl)**	**P_F_ (P_c_)**
**TNF**	**IE patients**	81 (75.7)	23 (21.5)	3 (2.8)	0.458	185 (86.4)	29 (13.6)	0.9036	0.344
**c.-1043C>A**	**controls**	128 (69.6)	52 (27.7)	5 (2.7)	(1.000)	307 (83.4)	61 (16.6)	(0.5697 to 1.433)	(1.000)
		**CC**	**CA**	**AA**	**P_χ_ (P_c_)**	**C**	**A**	**Odds ratio (95% Cl)**	**P_F_ (P_c_)**
**IL10**	**IE patients**	78 (59.1)	44 (33.3)	10 (7.6)	0.425	200 (75.8)	64 (24.2)	1.004	1.000
**c.-627C>A**	**controls**	104 (56.2)	72 (38.9)	9 (4.9)	(0.425)	280 (77.7)	90 (24.3)	(0.6949 to 1.452)	(1.000)
		**GG**	**GA**	**AA**	**P_χ_ (P_c_)**	**G**	**A**	**Odds ratio (95% Cl)**	**P_F_ (P_c_)**
**IL6**	**IE patients**	12 (10.1)	82 (68.9)	25 (21.0)	<0.0001	106 (44.5)	132 (55.5)	1.020	0.933
**c.471+870G>A**	**controls**	41 (22.2)	81 (43.8)	63 (34.0)	(<0.0003)	163 (44.1)	207 (55.9)	(0.7347 to 1.416)	(1.000)
		**GG**	**GA**	**AA**	**P_χ_ (P_c_)**	**G**	**A**	**Odds ratio (95% Cl)**	**P_F_ (P_c_)**
**IL6**	**IE patients**	31 (26.1)	68 (57.1)	20 (16.8)	0.113	130 (54.6)	108 (45.4)	1.142	0.4506
**c.-661A>G**	**controls**	65 (35.3)	83 (45.1)	36 (19.6)	(0.339)	213 (57.9)	155 (42.1)	(0.8219 to 1.586)	(1.000)
		**GG**	**GC**	**CC**	**P_χ_ (P_c_)**	**G**	**C**	**Odds ratio (95% Cl)**	**P_F_ (P_c_)**
**IL6**	**IE patients**	31 (26.0)	64 (53.8)	24 (20.2)	0.078	126 (52.9)	112 (47.1)	1.333	0.093
**c.-237C>G**	**controls**	71 (38.4)	80 (43.2)	34 (18.4)	(0.234)	222 (60.0)	148 (40.0)	(0.9597 to 1.852)	(0.279)

For IL1B, three different SNVs were analyzed. Allele and genotype distribution did not differ between patients and controls for IL1B c.-118C>T. The IL1B c.302-64 A allele was found frequently higher in the control group (37.3% vs. 31.7%; P_c_ = 0.612). The homozygote genotype AA was found in 13.5% of the control group, in contrast to 5.7% of the patient cohort, but a significant association could not be detected (P>0.05). The IL1B c.315 T allele showed a higher frequency in this group (32.2% vs. 26.2%) due to a higher heterozygote frequency in the patient group (49.0% vs. 34.1%). The Bonferroni corrected P-value did not confirm an association of the T allele with the patient cohort (P_c_ = 0.447).

Analysis of the three promotor variants of the TNF gene (c.-418G>A, c.-488G>A, c.-1043C>A) showed no differences in allele frequencies. For c.-488G>A and TNF c.-1043C>A, noticeable disparities can be shown regarding the genotype frequencies (c.-488G>A; GG IE patients 70.1% vs. GG controls 62.7%; c.-1043C>A: CC IE patients 75.7% vs. 69.9%).

Three different SNVs were analyzed in the IL6 gene. The intronic SNV c.471+870G>A showed no significant association with IE regarding the allele distribution. However, the genotype frequencies showed a markedly higher appearance of heterozygotes in patients (68.9% vs. 43.8%, P_c_<0.0003), whereas the frequency of homozygote genotypes AA and GG was significantly lower. IL6 c.-661A>G also did not differ significantly regarding the allelic distribution (A allele: controls 42.1%, patients: 45.4%), but again, the homozygote genotype of the G allele was noticeably higher in the control group (35.3% vs. 26.1%), whereas the heterozygote genotype was found more often in the patient group (57.1% vs. 45.1%). A significant difference between controls and patients could not be shown (P_c_ = 0.339). SNV IL6 c.-237C>G had a noticeably higher frequency of the minor allele in the patient cohort (C allele: 47.1% vs. 40.0%, P_c_ = 0.279). The genotypes GC was more frequent in the patient group (53.8% vs. 43.2%), but again, these frequencies did not reach statistical significance (P_c_ = 0.234).

Haploview analysis of haplotypes for IL1B, TLR4, TNF and IL6 did not reveal any associated haplotype with IE (data not shown).

### Association of SNVs with the IE-causing pathogen

In addition to the association study, the IE patient cohort was divided into four pathogen-associated subgroups: *Staphylococcus*, *Streptococcus*, *Enterococcus* and other IE cases. The allele frequencies were compared to determine a potential association of allele types with the causing pathogen (Table 2).

**Table 2:** Allele distribution and frequencies for pathogen-subgrouped patient cohort in comparison with controls P: P-value; P_c_: corresponding P-values after Bonferroni correction.

Subgroup		*Staphylococcus*spp.	*Streptococcus*spp.	*Enterococcus*spp.	others	controls		*Staphylococcus*spp.	*Streptococcus*spp.	*Enterococcus*spp.	others	controls
**Variation**	**IL1B c.-** **118C>T**						**TNF c.-418G>A**					
**Allele** **[n(%)]**	T	52 (65.0)	42 (72.4)	20 (71.4)	29 (69.1)	259 (70.0)	G	82 (97.6)	58 (96.7)	27 (90.0)	36 (90.0)	356 (96.2)
	C	28 (35.0)	16 (27.6)	8 (28.6)	13 (30.9)	111 (30.0)	A	2 (2.4)	2 (3.3)	3 (10.0)	4 (10.0)	14 (3.8)
	P (P_c_)	0.423 (1.000)	0.759 (1.000)	1.000 (1.000)	0.861 (1.000)		P (P_c_)	0.747 (1.000)	1.000 (1.000)	0.127 (0.381)	0.087 (0.261)	
**Variation**	**IL1B** **c.302-64G>A**						**TNF c.-488G>A**					
**Allele** **[n(%)]**	G	53 (66.2)	40 (69.0)	19 (67.9)	30 (71.4)	232 (62.7)	G	72 (85.7)	48 (80.0)	26 (86.7)	31 (77.5)	292 (78.9)
	A	27 (33.8)	18 (31.0)	9 (32.1)	12 (28.6)	138 (37.3)	A	12 (14.3)	12 (20.0)	4 (13.3)	9 (22.5)	78 (21.1)
	P (P_c_)	0.610 (1.000)	0.383 (1.000)	0.687 (1.000)	0.312 (0.936)		P (P_c_)	0.175 (0.525)	1.000 (1.000)	0.479 (1.000)	0.839 (1.000)	
**Variation**	**IL1B c.315C>T**						**TNF c.-1043C>A**					
**Allele** **[n(%)]**	C	55 (68.8)	40 (69.0)	17 (60.7)	29 (69.1)	273 (73.8)	C	73 (89.3)	49 (81.7)	26 (86.7)	37 (92.5)	307 (83.4)
	T	25 (31.2)	18 (31.0)	11 (39.3)	13 (30.9)	97 (26.2)	A	11 (10.7)	11 (18.3)	4 (13.3)	3 (7.5)	61 (16.6)
	P (P_c_)	0.405 (1.000)	0.430 (1.000)	0.184 (0.552)	0.581 (1.000)		P (P_c_)	0.510 (1.000)	0.712 (1.000)	0.800 (1.000)	0.171 (0.513)	
**Variation**	**IL6 c.471+870G>A**						**TLR4 c.776A>G**					
**Allele** **[n(%)]**	G	41 (46.5)	28 (42.4)	17 (44.7)	20 (43.5)	163 (44.1)	A	98 (92.4)	81 (92.1)	38 (90.5)	58 (96.7)	351 (94.9)
	A	47 (53.5)	38 (57.6)	21 (55.3)	26 (56.5)	207 (55.9)	G	8 (7.6)	7 (7.9)	4 (9.5)	2 (3.3)	19 (5.1)
	P (P_c_)	0.721 (1.000)	0.893 (1.000)	1.000 (1.000)	1.000 (1.000)		P (P_c_)	0.345 (0.690)	0.308 (0.616)	0.276 (0.552)	0.752 (1.000)	
**Variation**	**IL6 c.-661A>G**						**TLR4 c.1076C>T**					
**Allele** **[n(%)]**	G	47 (53.4)	38 (57.6)	22 (57.9)	23 (50.0)	213 (57.9)	C	98 (92.5)	81 (92.1)	38 (90.5)	58 (96.7)	351 (94.9)
	A	41 (46.6)	28 (42.4)	16 (42.1)	23 (50.0)	155 (42.1)	T	8 (7.5)	7 (7.9)	4 (9.5)	2 (3.3)	19 (5.1)
	P (P_c_)	0.473 (1.000)	1.000 (1.000)	1.000 (1.000)	0.345 (1.000)		P(P_c_)	0.345 (0.690)	0.308 (0.616)	0.276 (0.552)	0.752 (1.000)	
**Variation**	**IL6 c.-237C>G**						**SELE c.-19G>T**					
**Allele** **[n(%)]**	G	45 (51.1)	37 (56.1)	22 (57.9)	22 (47.8)	222 (60.0)	G	93 (89.4)	76 (90.5)	43 (89.6)	57 (91.9)	324 (87.6)
	C	43 (48.9)	29 (43.9)	16 (42.1)	24 (52.2)	148 (40.0)	T	11 (10.6)	8 (9.5)	5 (10.4)	5 (8.1)	46 (12.4)
	P (P_c_)	0.149 (0.447)	0.587 (1.000)	0.823 (1.000)	0.152 (0.456)		P (P_c_)	0.733 (0.733)	0.576 (0.576)	0.817 (0.817)	0.399 (0.399)	
**Variation**	**ICAM1 c.1405A>G**						**IL10 c.-627C>A**					
**Allele** **[n(%)]**	A	61 (56.5)	47 (54.6)	25 (54.3)	38 (59.4)	212 (57.3)	C	78 (74.5)	57 (71.3)	29 (76.3)	36 (81.8)	280 (77.7)
	G	47 (43.5)	39 (45.4)	21 (45.7)	26 (40.6)	158 (42.7)	A	24 (23.5)	23 (28.7)	9 (23.7)	8 (18.2)	90 (24.3)
	P (P_c_)	0.912 (0.912)	0.717 (0.717)	0.753 (0.753)	0.786 (0.786)		P (P_c_)	1.000 (1.000)	0.398 (0.398)	1.000 (1.000)	0.455 (0.455)	

None of the analyzed variants was found considerably more often in a pathogen-associated subgroup (P>0.05), but some variants showed a noticeable allele frequency in the subgroups. In the *Enterococcus* subgroup, a lower frequency of TNF c.-488G>A in comparison to the control group was shown (13.3% vs. 21.1%). In contrast, other variations showed a higher minor allele frequency in the *Enterococcus* subgroup in comparison to controls (e.g. IL1B c.315T 39.3% vs. 26.2%, TNF c.-418A 10.0% vs. 3.8%).

The subgroup composed of other organisms (culture-negative endocarditis, fungal species or more than one causative pathogen) showed noticeable allele frequencies in some cases. For example, IL1B c.302-64A showed a frequency of 28.6% in this subgroup compared to a considerably higher frequency of the A allele in controls (37.3%) (P_c_ = 0.936). In addition, IL10 c.-627A was found in 18.2% of patients in this subgroup, whereas the allele frequency in the control group was obviously higher (24.3%). The TNF c.-418A allele frequency was likewise increased in this subgroup (10.0% vs. 3.8%, P_c_ = 0.261). In addition, the IL6 c.-661A allele and c.-237G allele showed a high frequency in the subgroup of other pathogens compared with the control group (c.-661A 50% vs. 42.1%, P_c_ = 1.000 and c.-237C 52.2% vs. 40.0%, P_c_ = 0.456).

The *Staphylococcus* subgroup showed minor increased allele frequencies for the SNVs IL6 c.-661A (46.6% vs. 42.1%, P_c_ = 1.000) and c.-237C (48.9% vs. 40.0%, P_c_ = 0.447).

### Correlation between SNVs with CRP level, WBC counts and PCT level

To examine a potential association of SNVs with an altered inflammation in IE, genotype distributions were correlated with the inflammatory laboratory parameters CRP, WBC and PCT. In order to show potential influence of SNVs on inflammatory response, pre- and postoperative values for CRP, WBCs and PCT were examined (maximum two days before or after surgical intervention). Additionally, the differences in individual response in the course of heart valve replacement were calculated (quotient post−/preoperative value; Q_(post/pre)_).

Associations of preoperative biomarkers level might show hints to an altered reaction to pathogens. Furthermore, analysis of postoperative values or consideration of the quotient Q_(post/pre)_ could provide clues of an altered reaction to surgical intervention (e. g. mechanistically stress).

**Figure 1:**
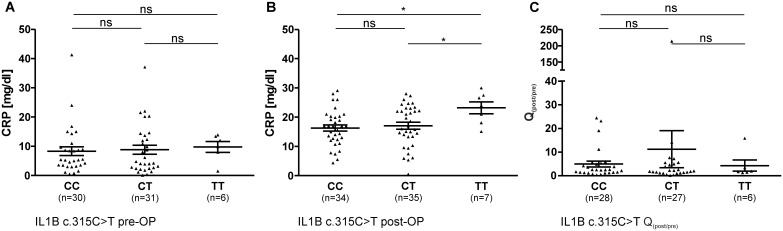
Pre- (A) and postoperative (B) CRP levels (mean ± SEM), as well as factorial change Q_(post/pre)_ of CRP (C) in the course of surgical intervention in patient sera in correlation with IL1B c.315C>T genotype (ns: not significant; * = P<0.05).

**Figure 2:**
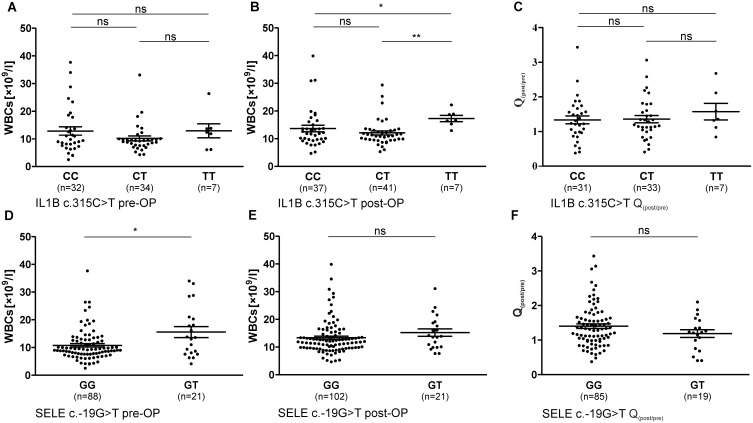
Pre- and postoperative WBC counts (mean ± SEM), as well as factorial change Q_(post/pre)_ of WBCs in the course of surgical intervention in patient sera (ns: not significant; * = P<0.05, ** = P<0.005). 2A–C: WBC counts in correlation with IL1B c.315C>T; A: Preoperative WBCs, B: Postoperative WBCs, C: Q_(post/pre)_ of CRP in the course of surgical intervention; 2E–G: WBC counts in correlation with SELE c.-19G>T; E: Preoperative WBCs, F: Postoperative WBCs, G: Q_(post/pre)_ of CRP in the course of surgical intervention.

**Figure 3:**
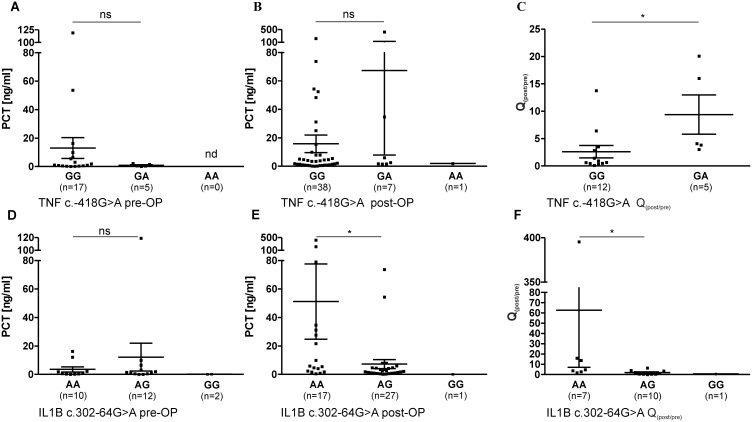
Pre- and postoperative PCT levels (mean ± SEM), as well as factorial change Q_(post/pre)_ of PCT in the course of surgical intervention in patient sera (ns: not significant; nd: not determined, * = P<0.05). 3A–C: PCT levels in correlation with TNF c.-418G>A; A: Preoperative PCT level, B: Postoperative PCT level, C: Q_(post/pre)_ of PCT in the course of surgical intervention; 3E–G: PCT levels in correlation with IL1B c.302-64G>A; A: Preoperative PCT level, B: Postoperative PCT level, C: Q_(post/pre)_ of PCT in the course of surgical operative intervention.

No relation between laboratory biomarkers and TLR4, IL10 and IL6 SNVs could be found (data not shown). The SNVs IL1B c.315C>T and c.302-64G>A, as well as SELE c.-19G>T and TNF c.-418G>A, can be associated with altered levels of CRP, WBC or PCT (Figures 1–3). The other SNVs belonging to TNF and IL1B were also not associated with alterations in these biomarkers.

IL1B c.315C>T was associated with altered CRP levels in patients (Figure 1). Preoperative values showed no differences between genotypes of c.315C>T (Figure 1A), but, in contrast, the mean CRP values of patients with genotype TT (23.19 mg/dl) were significantly higher after surgical intervention in comparison to patients with genotype CC (16.29 mg/dl, P = 0.012, P_c_ = 0.036) or CT (17.06 mg/dl, P = 0.040, P_c_ = 0.120) (Figure 1B). However, no significant differences concerning the Q_(post/pre)_ of CRP values in correlation to IL1B c.315C>T genotypes could be observed (Figure 1C).

In addition to higher CRP levels, postoperative WBC counts of patients with IL1B c.315C>T genotype TT (17.29×10^9^/l) were significantly higher than postoperative WBC counts of patients carrying the C allele (CC: 13.67×10^9^/l, P = 0.015, P_c_ = 0.045; CT: 12.09×10^9^/l, P = 0.002, P_c_ = 0.006) (Figure 2B). Preoperative WBC counts or factorial change between pre- and postoperative levels were not associated with IL1B c.315C>T genotype (Figures 2A, 2C).

Additionally, SELE c.-19G>T influenced WBC counts in patients group (Figures 2D–F). As shown in Figure 2D, preoperative WBC counts were significant higher in patients carrying the T allele (GG: 10.78×10^9^/l, GT: 15.59×10^9^/l; P = 0.040, P_c_ = 0.040). WBC counts after surgically intervention or Q_(post/pre)_ were not associated with SELE c.-19G>T (Figures 2E, 2F).

The PCT levels of the patient group were shown to be associated with SNVs TNF c.-418G>A and IL1B c.302-64G>A (Figure 3). As shown in figure 3A and B, TNF c.-418G>A did not influence pre- or postoperative levels of PCT in IE patients. However, it can be shown, that Q_(post/pre)_ of PCT in the course of surgical intervention is higher in patients carrying the A allele (GA: 9.39) than the Q_(post/pre)_ of patients with genotype GG (2.62, P = 0.018, P_c_ = 0.054) (Figure 3C).

IL1B c.302-64G>A influences postoperative PCT values, as well as the factorial enhancement of PCT, in the course of surgical intervention, as shown in Figure 3D–F. Preoperative PCT levels did not differ between IL1B c.302-64G>A genotypes (Figure 3D). However, patients with GA genotype showed significantly lower PCT levels after surgical intervention (Figure 3E) (AA: 51.18 ng/ml, AG: 7.25 ng/ml, P = 0.012, P_c_ = 0.036). In concordance, Q_(post/pre)_ was shown to be decreased in patients with the heterozygote genotype (AA: 62.6, AG: 1.9, P = 0.014, P_c_ = 0.042).

## Discussion

Inflammatory parameters are essential factors in the pathophysiology of IE. Genetic variations in cytokine genes have a wide research spectrum regarding other infectious diseases, but data suggesting an altered susceptibility to IE in association with genetic variations in candidate genes of inflammation are rare [8]. In this study, we analyzed a large number of SNVs in connection to IE, showing a potential influence of the SNVs IL1B c.315C>T, IL6 c.471+870G>A and IL6 c.-237C>G in association with IE. Although significance only remained after Bonferroni correction in the case of IL6 c.471+870G>A, these three variants remain potential candidates for influencing susceptibility to IE. For genetic variants in TLR4 and IL10, no associations to IE susceptibility were observed in this study.

IL1B c.315C>T showed a higher frequency of the T allele in the patient cohort (32.2% vs. 26.2%, p = 0.149, P_c_ = 0.447). Some studies assume that IL1B c.315C>T influences IL1B protein expression, whereas others were not able to support this hypothesis [18], [19]. Additionally, the influence of IL1B c.315C>T to the inflammatory response was shown throughout a decreased CRP concentration in sera of healthy individuals in association with genotype TT [20]. In our study group, there were significantly higher postoperative CRP levels and WBC counts associated with c.315 TT. The interrelationship of IL1B genetic variants and CRP has previously been discussed in the context of other diseases [21]. Therefore, this genetic variation of IL1B is possibly associated with an altered postoperative inflammatory response. Increased postoperative CRP levels and WBC counts point to an extended inflammation of patients with genotype TT and, as previously shown, abnormal values of CRP or WBC can be associated with increased mortality of IE patients [16]. In addition, IL1B c.302-64G>A heterozygote genotype was associated with a decreased factorial enhancement of PCT in the course of surgical intervention, although this SNV was not associated with IE. Interestingly, IL1B c.315 T allele was detected more often in the *Enterococcus* subgroup. A pathogen-dependent association of SNVs with IE susceptibility is conceivable, but remains unsolved so far.

No differences in genotype or allele frequencies between IE patients and controls can be observed for SELE c.-19G>T. However, regarding the preoperative level of laboratory parameters, a significantly higher WBC count was shown in association with the genotype GT. Abnormal WBC counts in IE were associated with mortality [16]. Therefore, this observation could hint at an association of SELE c.-19G>T genotype to alterations concerning the individual inflammatory response to pathogens during IE disease progression.

Three SNVs in the promotor region of TNF gene were analyzed in this association study. Although an association of TNF c.-488G>A with sepsis or severity of sepsis was demonstrated in many studies, Jessen et al. also failed to confirm an association with gram-negative sepsis [22]–[25]. Our data showed no influence of TNF promotor variants c.-418G>A, c.-488G>A or c.-1043C>A on IE susceptibility. Some studies revealed altered protein levels in concordance with TNF promotor SNVs, whereas others did not confirm this association [26]. The correlation of laboratory parameters revealed an association of TNF c.-418G>A with the individual factorial change Q_(post/pre)_ of PCT in the course of surgical heart valve replacement. Whether or not this observation regarding altered PCT in linkage to TNF c.-418G>A and IL1B c.302-64G>A shows an influence towards alterations in the inflammatory response remains unsolved so far.

The IL6 variants are shown to be particularly noteworthy. IL6 c.471+870 GA genotype was significantly associated with IE patients. The frequency of heterozygote genotype of the other two IL6 variants was also noticeably higher in IE patients, although differences failed to be statistically significant. Taken together, the influence of IL6 variants could influence IE responsiveness of individuals. Tischendorf et al. demonstrated an influence of c.-237C>G on IL6 plasma concentration in patients with severe sepsis, whereby *ex* *vivo* LPS stimulation of IL6 was pronounced in patients with CC or CG genotype [27]. IL6 gene variants were found to be associated with higher systolic blood pressure [28]. Although the effect of IL6 gene variants on blood pressure seems to be modest, the *in* *vivo* affection in IE cannot be assessed. Alterations in blood pressure could promote injuries to the endocardium, serving as nidus for bacterial adhesion to endothelial cells. A weak association of c.-237C>G with an increased mortality in the patient cohort with severe sepsis was demonstrated [29]. The IL6 gene variant c.471+870G>A is an intron gene variant, and only marginal information is accessible to date. However, the heterozygote genotype of this genetic variant was detected more often in IE patients. It was described, that this SNV includes a CpG site, but influence on IL6 expression in inflammatory response is not known up to now [30]. Therefore, we assume that this SNV is probably linked to another variant which potentially has a noticeable influence on IL6 response in the pathogenesis of IE. Further investigation into different haplotypes of IL6 could approve this hypothesis. A quantity of other SNV is located downstream of this SNV, but to our knowledge, showing no suspicion to be linked to other infectious diseases so far. Based on the data of this study, a detailed analysis of this region is intended in a future prospective study.

Certainly, our study has some limitations. It was not possible to compare all IE patients with regard to all genetic variations, due to limited patient material. To overcome this limitation, each gene was analyzed in an individual patient cohort, not influencing the study results because strong associations were shown independently from patient characteristics. Furthermore, the IE patients show a high heterogeneity regarding infection severities or stages of antibiotic treatment. Additionally, our cohort merely consists of patients, which needs a surgically intervention in the course of IE disease progression. Overall, surgical intervention in course of IE is required in up to 50% of all IE cases [31]. Although even uncomplicated IE cases would be certainly interesting, the IE cohort in this study represents a homologue group of IE patients with definite IE cases.

In conclusion, the results of our study demonstrate a relationship of host genetics and IE. The IL6 c.471+870G>A genotypes showed a significant association with an increased IE susceptibility. Additional observations in this study gave hints, that advanced investigations with extended haplotypes could reveal further associations. Our study provides several hints towards an altered inflammation in IE disease progression, which should be analyzed in further investigations.

## Supporting Information

Table S1 Patient and control characteristics.
(DOCX)

Table S2 PCR primers, annealing temperature (T_AN_), amplificate length, MgCl_2_ concentration and endonucleases for RFLP-analysis of gene variants. Reference sequence (RefSeq) refers to NCBI dbSNP.
(DOCX)
