# Supplementary material for: Genetic Variants in Genes of the Inflammatory Response in Association with Infective Endocarditis
Source: PLoS One. 2014 Oct 9;9(10):e110151. doi: 10.1371/journal.pone.0110151 (PMC4192365; doi:10.1371/journal.pone.0110151)
Supplement: Table S1 — Patient and control characteristics. (DOCX) [file pone.0110151.s001.docx]

**Supplemental Data Table S1**

| **Controls** |  |  |  |  |
| --- | --- | --- | --- | --- |
| *Age* | *n* | *Years (mean)* | *±SD* | *range* |
| Male | 130 | 54.2 | 10.7 | 15-67 |
| Female | 55 | 55.5 | 11.3 | 27-68 |
| **IE patients IL1B** | |  |  |  |
| *Age* | *n* | *Years (mean)* | *±SD* | *range* |
| Male | 75 | 58.8 | 14.9 | 15-81 |
| Female | 29 | 63.3 | 14.1 | 28-84 |
| **IE patients TLR** | |  |  |  |
| *Age* | *n* | *Years (mean)* | *±SD* | *range* |
| Male | 104 | 58.0 | 14.7 | 28-84 |
| Female | 44 | 63.7 | 14.8 | 14-81 |
| **IE patients SELE** | |  |  |  |
| *Age* | *n* | *Years (mean)* | *±SD* | *range* |
| Male | 105 | 58.0 | 14.7 | 14-81 |
| Female | 44 | 64.9 | 14.4 | 28-84 |
| **IE patients ICAM1** | |  |  |  |
| *Age* | *n* | *Years (mean)* | *±SD* | *range* |
| Male | 106 | 59.3 | 15.2 | 14-81 |
| Female | 46 | 59.2 | 15.0 | 14-84 |
| **IE patients TNF** | |  |  |  |
| *Age* | *n* | *Years (mean)* | *±SD* | *range* |
| Male | 74 | 60.3 | 12.1 | 16-78 |
| Female | 35 | 62.7 | 15.4 | 28-84 |
| **IE patients IL10** | |  |  |  |
| *Age* | *n* | *Years (mean)* | *±SD* | *range* |
| Male | 90 | 57.8 | 14.1 | 14-81 |
| Female | 42 | 62.6 | 14.6 | 28-84 |
| **IE patients IL6** | |  |  |  |
| *Age* | *n* | *Years (mean)* | *±SD* | *range* |
| Male | 83 | 58.5 | 14.3 | 14-78 |
| Female | 36 | 65.3 | 14.3 | 28-84 |
