# Supplementary material for: Genetic Variants in Genes of the Inflammatory Response in Association with Infective Endocarditis
Source: PLoS One. 2014 Oct 9;9(10):e110151. doi: 10.1371/journal.pone.0110151 (PMC4192365; doi:10.1371/journal.pone.0110151)
Supplement: Table S2 — PCR primers, annealing temperature (TAN), amplificate length, MgCl2 concentration and endonucleases for RFLP-analysis of gene variants. Reference sequence (RefSeq) refers to NCBI dbSNP. (DOCX) [file pone.0110151.s002.docx]

**Supplemental Data Table S2**

| **Gene/rs-number**  **(RefSeq)** | **forward primer (5'…3') reverse primer (5'…3')** | **T_AN_**  **[°C]** | **product** | **MgCl_2_ (mM)** | **enzyme (/reaction)** | **allele** | **products** |
| --- | --- | --- | --- | --- | --- | --- | --- |
| IL1β rs1143627 | GCTTCCACCAATACTCTTTTCCCCTT | 59 | 173 bp | 2.5 | *Alu*I | T | 98 bp |
| (NM_000576.2:c.-118C>T) | CCCAGAGCAGCCTGTTGTGCC |  |  |  | (5.0 U) |  | 75 bp |
| IL1β rs1143633 | GTTGTCATCAGACTTTGACC | 58 | 250 bp | 2.5 | *Aci*I | G | 192 bp |
| (NM_000576.2:c.302-64G>A) | TTCAGTTCATATGGACCAGA |  |  |  | (3.0 U) |  | 58 bp |
| IL1β rs1143634 | GTTGTCATCAGACTTTGACC | 58 | 250 bp | 2.5 | *Taq*I | C | 136 bp |
| (NM_000576.2:c.315C>T) | TTCAGTTCATATGGACCAGA |  |  |  | (2.0 U) |  | 114 bp |
| TLR4 rs4986790 | GATTAGCATACTTAGACTACTACCTCCATG | 55 | 249 bp | 2.5 | *Nco*I | G | 223 bp |
| (NM_003266.3:c.776A>G) | GATCAACTTCTGAAAAAGCATTCCCAC |  |  |  | (2.0 U) |  | 26 bp |
| TLR4 rs4986791 | GGTTGCTGTTCTCAAAGTGATTTTGGGAGAA | 65 | 407 bp | 2.5 | *Hinf*I | T | 378 bp |
| (NM_003266.3:c.1076C>T) | ACCTGAAGACTGGAGAGTGAGTTAAATGCT |  |  |  | (2.0 U) |  | 29 bp |
| SELE rs1805193 | ATTTTGTGTTTTATCTCCCCAG | 52 | 188 bp | 1.5 | *MseI* | T | 137 bp |
| (NM_000450.2:c.-19G>T) | CCCCAGACAAGCAAGGAT |  |  |  | (1.0 U) |  | 51 bp |
| ICAM rs5498 | ATTCCCAGCAGACTCCAA | 60 | 272 bp | 2.5 | *BstUI* | G | 169 bp |
| (NM_000201.2:c.1405A>G) | GGAGGATACAACAGGCGG |  |  |  | (2.0 U) |  | 103 bp |
| TNF rs361525 | TCAGTGGCCCAGAAGACCCCCCTCGGAACC | 65 | 278 bp | 2.5 | *Msp*I | G | 248 bp |
| (NM_000594.3:c.-418G>A) | GTCTGCTGGCTGGGTGTGCCAACAACTG |  |  |  | (2.5 U) |  | 30 bp |
| TNF rs1800629 | AAATGGAGGCAATAGGTTTTGAGGGCCATG | 59 | 348 bp | 2.5 | *Nco*I | G | 322 bp |
| (NM_000594.3:c.-488G>A) | GTCTGCTGGCTGGGTGTGCCAACAACTG |  |  |  | (1.0 U) |  | 26 bp |
| TNF rs1800630 | GCAGGGGAAGCAAAGGAGAAGCTGAGAACA | 54 | 229 bp | 3.5 | *Bsa*AI | A | 200 bp |
| (NM_000594.3:c.-1043C>A) | GGCCCTCTACATGGCCCTGTCTTCGTTACG |  |  |  | (2.5 U) |  | 29 bp |
| IL10 rs1800872 | AGCTGAAGAGGTGGAAAC | 54 | 250 bp | 2.5 | *Rsa*I | A | 165 bp |
| (NM_000572.2:c.-627A>C) | CCAACCTGGGATGAATAC |  |  |  | (2.0 U) |  | 85 bp |
| IL6 rs2069845 | TCCAGGACCACACTTGGA | 54 | 184 bp | 2.5 | *Msp*I | G | 96 bp |
| (NM_000600.3:c.471+870G>A) | GGGTTCTTCAGTGGCCTCTA |  |  |  | (1.5 U) |  | 88 bp |
| IL6 rs1800797 | AAAAAGGAGTCACACACTCCAC | 62 | 535 bp | 2.5 | *Fok*I | A | 464 bp |
| (NM_000600.3:c.-661A>G) | GGGCTGATTGGAAACCTTATTAAGA |  |  |  | (1.0 U) |  | 71 bp |
| IL6 rs1800795 | AAAAAGGAGTCACACACTCCAC | 62 | 535 bp | 2.5 | *Lwe*I | G | 474 bp |
| (NM_000600.3:c.-237C>G) | GGGCTGATTGGAAACCTTATTAAGA |  |  |  | (4.0 U) |  | 61 bp |
